# Elevated mitochondrial DNA copy number in euthyroid individuals with impaired peripheral sensitivity to thyroid hormones

**DOI:** 10.3389/fendo.2025.1635820

**Published:** 2025-09-19

**Authors:** Li Qin, Tingting Huang, Danmei Zhang, Guochao Li, Liqin Wei, Juan Liu

**Affiliations:** Division of Geriatric Endocrinology, The First Affiliated Hospital with Nanjing Medical University, Nanjing, China

**Keywords:** euthyroid individuals, FT3/FT4 ratio, mitochondrial DNA copy number (mtDNAcn), thyroid hormone sensitivity, thyroid feedback quantile-based index (TFQI), thyroid-stimulating hormone index (TSHI), thyrotrophic thyroxine resistance index (TT4RI)

## Abstract

**Background:**

The role of thyroid hormone sensitivity in metabolic and neoplastic diseases is well recognized, however, its association with mitochondrial DNA copy number (mtDNAcn) in euthyroid individuals remains unclear.

**Objective:**

This cross-sectional study aimed to explore the relationship between thyroid hormone sensitivity and mtDNAcn in euthyroid individuals.

**Methods:**

We recruited 350 hospitalized individuals with normal thyroid function between December 2020 and August 2022. Peripheral blood samples and clinical parameters were collected. Relative mtDNAcn levels were quantified by qPCR from peripheral blood samples. Peripheral thyroid hormone sensitivity was evaluated through the free triiodothyronine to free thyroxine (FT3/FT4) ratio, and central sensitivity was assessed using the thyrotrophic thyroxine resistance index (TT4RI), thyroid-stimulating hormone index (TSHI), and thyroid feedback quantile-based index (TFQI).

**Results:**

Significant differences in age and sex were observed between groups with lower and higher mtDNAcn. Multivariate linear regression analysis showed a negative correlation between mtDNAcn and the FT3/FT4 ratio after adjusting for confounders such as age, sex, BMI, alcohol consumption, smoking history, hypertension, diabetes, and hyperlipidemia. This negative correlation remained significant in subgroups of younger adults, females, normal-weight individuals, drinkers, non-smokers, and those with hypertension. No significant relationships were found between mtDNAcn and TSHI, TT4RI, or TFQI after adjusting for confounding factors.

**Conclusion:**

Reduced peripheral thyroid hormone sensitivity is linked to elevated mtDNAcn in euthyroid individuals, with variations based on age, sex, BMI, smoking, and hypertension.

## 1 Introduction

Thyroid hormones, particularly tetraiodothyronine (T4) and triiodothyronine (T3), are pivotal in regulating metabolic homeostasis in the body (1). These hormones influence various metabolic pathways, including basal metabolic rate, fat, protein, and carbohydrate metabolism, thereby affecting energy expenditure, body weight, and blood glucose levels. Dysfunctional thyroid activity can have detrimental health effects. Moreover, alterations in thyroid hormone sensitivity may contribute to metabolic disorders, even in euthyroid population, leading to conditions such as cardiovascular diseases, type 2 diabetes, and obesity (2–6). Thyroid hormone sensitivity describes how tissues react to thyroid hormones, which in turn affects metabolism. While serum levels of thyroid-stimulating hormone (TSH), free tetraiodothyronine (FT4), and free triiodothyronine (FT3) are commonly used to assess thyroid function, thyroid hormone sensitivity provides a more comprehensive understanding of thyroid function than hormone levels alone (7–10). Indicators of thyroid hormone sensitivity include the FT3/FT4 ratio, the thyroid-stimulating hormone index (TSHI), the thyrotrophic thyroxine resistance index (TT4RI), and the thyroid feedback quantile-based index (TFQI) (11–13). The FT3/FT4 ratio is an indicator of peripheral sensitivity to thyroid hormones and has been linked to various health outcomes, including blood glucose variability, handgrip strength, and all-cause mortality (14–16). TSHI, TFQI, and TT4RI represent central thyroid hormones sensitivity and have been associated with factors such as vitamin D levels, osteoarthritis risk, and prediabetes risk (5, 8, 17). In recent years, thyroid hormone sensitivity has emerged as a key focus in research on metabolic health in euthyroid populations.

Mitochondria, semi-autonomous organelle in most eukaryotic cells, play essential roles in cellular functions such as metabolism, energy production, growth, apoptosis, and signaling (18, 19). Mitochondrial DNA (mtDNA), a unique circular double-stranded genome, encodes RNAs and proteins critical for mitochondrial function (20). Mitochondrial DNA copy number (mtDNAcn) in peripheral blood can be quantified using quantitative real-time polymerase chain reactions (qPCR), serving as an indirect biomarker of mitochondrial function that is widely used in clinical research (21). Changes in mtDNAcn have been linked to various diseases, with mitochondrial dysfunction serving as a biomarker of aging. Reduced mtDNAcn is associated with aging and poor health outcomes (22–24). Our previous studies have found lower mtDNAcn levels in patients with frailty and cognitive frailty (25, 26). Additionally, observational studies suggested that decreased mtDNAcn was linked to an increased risk of metabolic disorders and all-cause dementia (27–30). Three large prospective studies have reported an independent association between mtDNAcn and the incidence of cardiovascular disease (31). Furthermore, low mtDNAcn in peripheral blood has been linked to an increased future risk of mortality (32). These findings have prompted further research into the relationship between mtDNAcn and various systemic diseases, including thyroid-related disorders.

Recent research has suggested that alterations in thyroid hormone sensitivity may influence disease progression by affecting mitochondrial dynamics (33–35). However, the underlying mechanism in euthyroid individuals remains unclear. Most previous research has focused on changes in mtDNAcn in individuals with thyroid dysfunction, such as thyroiditis or thyroid cancer. For example, patients with papillary thyroid carcinoma (PTC) show significantly elevated mtDNAcn in their tissues, which correlates with tumor aggressiveness (36). Conversely, individuals with hypothyroidism may experience a reduction in peripheral mtDNAcn due to oxidative stress accumulation (37, 38). Nevertheless, there is still limited evidence regarding whether thyroid hormone sensitivity, as opposed to hormone levels themselves, affects mtDNAcn in euthyroid populations.

Several studies indicates that thyroid hormones directly regulate mitochondrial biogenesis and function through both genomic and non-genomic pathways. Chainy GBN et al. proposed that T3 binds to nuclear thyroid receptors (TRα/β), activating PGC-1α signaling, which in turn stimulates TFAM-mediated mtDNA replication and respiratory chain complex synthesis (39). Clinically, alterations in mtDNAcn have been observed in thyroid dysfunction: hyperthyroidism increases mtDNAcn due to enhanced oxidative phosphorylation demand, whereas hypothyroidism decreases mtDNAcn through the accumulation of reactive oxygen species. Moreover, a recent study has shown a significant inverse correlation between fT4 levels and mtDNAcn in male newborns, suggesting a sex-specific influence of thyroid hormones on mitochondrial DNA replication (40). However, the influence of tissue-specific sensitivity to thyroid hormones on mtDNAcn remains unexplored. Furthermore, while existing studies have primarily focused on overt thyroid dysfunction, no research has examined whether peripheral or central thyroid hormone sensitivity indices correlate with mtDNAcn. Therefore, this study aims to explore the potential relationship between mtDNAcn and thyroid hormone sensitivity in euthyroid individuals based on clinical data. Additionally, subgroup analyses were conducted to explore how variations in factors such as age, sex, and body mass index (BMI) may impact thyroid hormone sensitivity and mtDNAcn.

## 2 Methods

### 2.1 Participants

**Figure 1:**
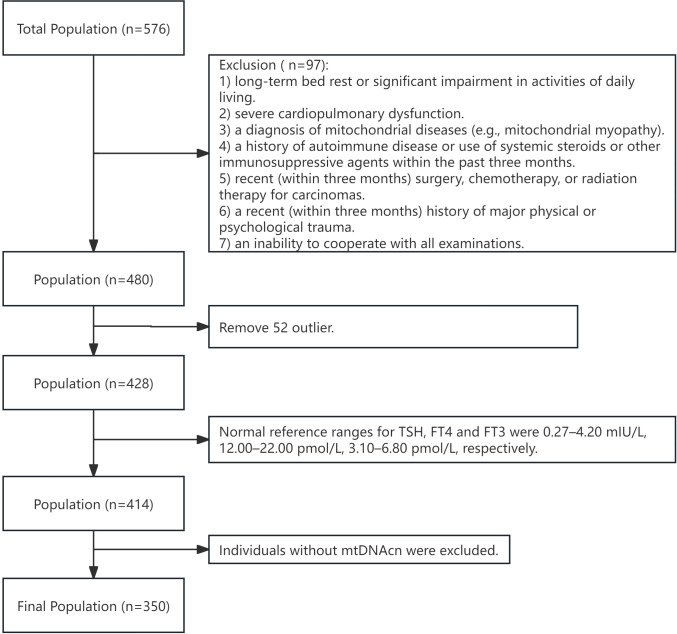
Flow chart of participants selection. mtDNAcn, mitochondrial DNA copy number.

The study was conducted at Jiangsu Province Hospital from October 2020 to August 2022 (**Figure 1**). Inclusion criteria included: 1) the ability to undergo all necessary tests; 2) stable underlying conditions controlled by medication (e.g., diabetes, hypertension, coronary heart disease). Exclusion criteria were: 1) long-term bed rest or significant limitations in daily living; 2) severe cardiopulmonary dysfunction; 3) a history of mitochondrial diseases (e.g., mitochondrial myopathy); 4) autoimmune disorders or recent use of systemic steroids or immunosuppressants within the last three months; 5) recent surgery, radiation therapy, or chemotherapy for cancer within the past three months; 6) significant physical or psychological trauma in the last three months; 7) inability to cooperate with the necessary examinations; 8) thyroid dysfunction (i.e., overt or subclinical hypothyroidism or hyperthyroidism), a history of thyroid surgery, thyroid disease, or medications affecting thyroid function. Additionally, subjects with extreme outlying data, abnormal FT3, FT4 or TSH levels, or those lacking mtDNAcn data were excluded. The study was approved by the Ethical Committee of Jiangsu Province Hospital (2024-SR-087, 2019-NT-48), and written informed consent was obtained from all participants.

### 2.2 Data collection

Clinical coordinators used pre-designed questionnaires to gather demographic and clinical data, including age, sex, drinking and smoking history, and a history of diabetes, hyperlipidemia, and hypertension. Trained nurses measured participants’ height and weight in a quiet setting. BMI was calculated using the formula: BMI=weight (kg)/height^2^ (m^2^). Venous blood samples were collected after at least 10 hours of fasting to measure FT3, FT4, TSH, serum glucose, glycohemoglobin, high-density lipoprotein (HDL), low-density lipoprotein (LDL), triglyceride (TG), total cholesterol (TC), serum uric acid (UA), and 25-hydroxyvitamin-D (VIT-D). The triglyceride-glucose (TyG) index, a simple predictor of metabolic disorders, was calculated as follows: Ln [TG (mg/dL) × FPG (mg/dL)/2] (41).

### 2.3 Indices of thyroid hormone sensitivity

Several indices were employed to assess thyroid hormone sensitivity, including the FT3/FT4 ratio, TT4RI, TSHI, and TFQI. The following formulas were used to calculate the indices:


FT3/FT4 ratio=FT3 (pmol/L)/FT4 (pmol/L)

TSHI=Ln TSH (mIU/L)+0.1345×FT4 (pmol)

(11)


TT4RI=FT4 (pmol/L)×TSH (mIU/L)

(12)


TFQI=cdfFT4-(1-cdfTSH)

(13)

The FT3/FT4 ratio reflects peripheral thyroid sensitivity, with higher ratios indicating enhanced peripheral sensitivity. TSHI, TT4RI, and TFQI assess central thyroid sensitivity, linked to hypothalamic-pituitary feedback mechanisms. For TSHI and TT4RI, lower values suggest greater central sensitivity. TFQI ranges from -1 to +1, where negative values indicate heightened central sensitivity and positive values suggest reduced sensitivity.

### 2.4 Total DNA extraction from peripheral blood

Blood samples were collected after a minimum of 10 hours of fasting using heparinized vacutainer tubes. A total of 200 μL of blood was used for genomic DNA extraction, employing the FastPure^®^ Blood DNA Isolation Mini Kit V2 (Vazyme, China) according to the manufacturer’s protocol. Briefly, 400 μL of ACK lysis buffer was added to the blood sample and centrifuged at 10,000 rpm for 1 minute at room temperature (RT). The pellet was resuspended in 200 μL PBS, followed by the addition of 20 μL Proteinase K and 200 μL Buffer BCL. The mixture was incubated at 56°C for 10 minutes with intermittent inversion. After adding 200 μL absolute ethanol, the lysate was transferred to a FastPure gDNA Mini Column and centrifuged at 12,000 rpm for 1 minute. The column was washed sequentially with 500 μL Buffer WA and 700 μL Buffer WB, each followed by centrifugation at 12,000 rpm for 30 seconds. After centrifuging at 12,000 rpm for 2 minutes to remove residual ethanol, DNA was eluted with 75 μL of preheated Elution Buffer (56°C) and quantified. The purified DNA was stored at −20°C.

### 2.5 Quantitative real-time polymerase chain reactions

The mtDNAcn was quantified using qPCR with the SYBR Green method. Each reaction had a total volume of 10 μL, containing 2 μL genomic DNA and 5 μL ChamQ Universal SYBR qPCR Master Mix (Vazyme, China). The thermal cycling conditions were as follows: 1 cycle at 95°C for 30 seconds, followed by 40 cycles of 95°C for 10 seconds and 60°C for 30 seconds. The relative mtDNAcn was quantified by calculating the ratio of mitochondrial DNA (mt-ND1, mitochondrial-NADH dehydrogenase subunit 1) to nuclear DNA (β-actin). Data analysis followed the 2−ΔΔCT method. The primers used for amplification were listed below:

**Table:** Primer sequences used for qPCR.

Gene	Forward primer (5’-3’)	Reverse primer (5’-3’)
MT-ND-1	CACTCACATCACAGCGCTAA	GGATTATGGATGCGGTTGCT
β-actin	ATTGGCAATGAGCGGTTCCGC	CTCCTGCTTGCTGATCCACATC

### 2.6 Statistical analysis

Data analysis was performed using SPSS (version 25.0) and R (version 4.2.1). The normality of the data was evaluated using skewness, kurtosis, the Shapiro – Wilk test, histograms, and Q-Q plots. Continuous variables were presented as mean ± standard deviation (SD), and categorical variables as percentages (%). Relative mtDNAcn levels were divided into two groups:< 0.96 and ≥ 0.96. Group comparisons were employed using appropriate statistical tests (e.g., t-test, Mann-Whitney U test, or chi-square test) based on the data distribution. Correlations between mtDNAcn and thyroid hormone sensitivity variables were analyzed within subgroups categorized by factors such as age, sex, BMI, alcohol consumption, smoking status, and the presence of diabetes, hypertension, or hyperlipidemia. Multivariate linear regression was conducted to explore the relationship between thyroid hormone sensitivity and mtDNAcn. Partial regression plots were used to examine the independent association between thyroid hormone sensitivity variables and mtDNAcn, with further subgroup analyses. Using G*Power software, the effect size (f²) was calculated from the adjusted R² of the final multiple linear regression model. With a significance level (α) of 0.05 and a model containing 9 variables (1 primary predictor and 8 adjusted covariates), the *post-hoc* analysis confirmed that the statistical power exceeded 0.80, meeting the conventional threshold. Data visualization was performed using the ggplot2 package in R. A *P*-value below 0.05 was regarded as statistically significant.

## 3 Results

### 3.1 Baseline characteristic and grouping

**Table 1** summarized the baseline characteristics of the 350 euthyroid participants included in this study, comprising 257 males (73.4%) and 93 females (26.6%), with a mean age of 62.15 ± 0.78 years and an average BMI of 24.83 ± 0.18 kg/m^2^. The prevalence of comorbidities was as follows: diabetes (n=141, 40.3%), hypertension (n=163, 46.6%), and hyperlipidemia (n=51, 14.6%). Based on relative mtDNAcn, participants were divided into two groups: mtDNAcn< 0.96 (n=175) and mtDNAcn ≥ 0.96 (n=175). The group with higher mtDNAcn showed a greater proportion of males and younger individuals. No statistically significant differences were observed between the groups in terms of BMI, smoking and alcohol consumption, metabolic comorbidities (diabetes, hypertension, hyperlipidemia), or laboratory measures including glycohemoglobin, serum glucose, lipid profile (TC, TG, LDL, HDL), VIT-D, UA, TyG index, or thyroid-related indicators (FT3, FT4, TSH, FT3/FT4 ratio, TSHI, TT4RI, TFQI).

**Table 1:** Characteristics of study population.

Characteristics	Total	Relative level of mtDNAcn		*P*-value
		< 0.96	≥ 0.96	
No.	350	175	175	
Sex (%)				0.04
Male	73.40	78.30	68.60	
Female	26.60	21.70	31.40	
Smoking (%)				0.20
Yes	49.10	52.60	45.70	
No	50.90	47.40	54.30	
Drinking (%)				0.24
Yes	50.00	53.10	46.90	
No	50.00	46.90	53.10	
Diabetes (%)				0.10
Yes	40.30	36.00	44.60	
No	59.70	64.00	55.40	
Hypertension (%)				0.34
Yes	46.60	49.10	44.00	
No	53.40	50.90	56.00	
Hyperlipidemia (%)				0.88
Yes	14.60	14.90	14.30	
No	85.40	85.10	85.70	
Age (years)	62.15 ± 0.78	63.74 ± 1.15	60.57 ± 1.06	0.04
BMI (kg/m^2^)	24.83 ± 0.18	24.62 ± 0.26	25.05 ± 0.26	0.36
GLU (mmol/L)	5.55 ± 0.09	5.62 ± 0.15	5.48 ± 0.13	0.72
Glycohemoglobin (%)	6.35 ± 0.08	6.38 ± 0.12	6.31 ± 0.11	0.70
TC (mmol/L)	4.42 ± 0.06	4.35 ± 0.08	4.50 ± 0.08	0.20
TG (mmol/L)	1.76 ± 0.07	1.78 ± 0.12	1.74 ± 0.09	0.50
HDL (mmol/L)	1.11 ± 0.01	1.10 ± 0.02	1.13 ± 0.02	0.41
LDL (mmol/L)	2.63 ± 0.04	2.56 ± 0.05	2.70 ± 0.06	0.08
VIT-D (nmol/L)	60.47 ± 1.78	63.04 ± 2.80	58.01 ± 2.21	0.43
UA (μmol/L)	352.00 ± 5.58	355.89 ± 7.38	349.56 ± 8.38	0.58
TyG	8.74 ± 0.04	8.74 ± 0.06	8.74 ± 0.05	0.78
FT3 (pg/mL)	4.56 ± 0.03	4.57 ± 0.05	4.55 ± 0.04	0.89
FT4 (ng/dL)	16.53 ± 0.11	16.54 ± 0.16	16.52 ± 0.15	0.95
TSH (mIU/L)	2.04 ± 0.05	1.99 ± 0.07	2.09 ± 0.07	0.25
FT3/FT4	0.28 ± 0.01	0.28 ± 0.01	0.28 ± 0.01	0.73
TSHI	2.84 ± 0.03	2.81 ± 0.04	2.87 ± 0.04	0.27
TT4RI	33.54 ± 0.78	32.70 ± 1.10	34.38 ± 1.11	0.24
TFQI	-0.09 ± 0.02	-0.10 ± 0.02	-0.08 ± 0.02	0.47
Relative level of mtDNAcn	1.05 ± 0.03	0.60 ± 0.02	1.50 ± 0.04	<0.0001

Categorical data were presented as percentages (%) and continuous variables were expressed as mean ± standard deviation (SD). Statistical differences between groups were assessed using Chi-square test, Student’s t-tests, or Mann–Whitney U tests, as appropriate. Statistical significance was defined at *P*-value< 0.05. mtDNAcn, mitochondrial DNA copy number; BMI, body mass index; GLU, serum glucose; TC, total cholesterol; TG, triglyceride; HDL, high-density lipoprotein; LDL, low-density lipoprotein; VIT-D, 25-hydroxyvitamin D; UA, serum uric acid; FT3, free triiodothyronine; FT4, free thyroxine; TSH, thyroid-stimulating hormone; TSHI, TSH index; TT4RI, thyrotrophic thyroxine resistance index; TFQI, thyroid feedback quantile-based index.

### 3.2 Association between mtDNAcn and thyroid hormone sensitivity

Correlation analyses were performed within subgroups stratified by age, sex, BMI, smoking status, alcohol use, and comorbidity (**Table 2**). A significant inverse correlation between the FT3/FT4 ratio and mtDNAcn was identified in younger individuals, while this association was absent in the older participants. No correlations between mtDNAcn and central sensitivity indices (TSHI, TT4RI, TFQI) in any age subgroup. Among non-smokers, a significant negative correlation was also observed between FT3/FT4 and mtDNAcn, which was not showed in smokers. Central sensitivity indices again showed no significant association with mtDNAcn regardless of smoking status. A similar pattern was observed in participants with hypertension, where an inverse association between FT3/FT4 and mtDNAcn was noted only in the hypertensive group. In the hyperlipidemia subgroup, mtDNAcn showed a significant positive correlation with TSHI, although FT3/FT4, TT4RI, and TFQI remained no association. In contrast, no statistically significant associations were observed in subgroups categorized by sex, BMI, alcohol consumption, or diabetes status.

**Table 2:** Associations between mtDNAcn and thyroid hormone sensitivity.

Variables	FT3/FT4		TSHI		TT4RI		TFQI	
	*r*	*p*	*r*	*p*	*r*	*p*	*r*	*p*
Age								
younger adult	-0.17	**0.025**	0.05	0.52	0.04	0.62	0.03	0.69
older adult	-0.06	0.46	0.06	0.43	0.07	0.38	0.11	0.15
Sex								
males	-0.05	0.46	0.05	0.44	0.04	0.54	0.07	0.25
females	-0.11	0.32	0.11	0.29	0.09	0.37	0.14	0.22
BMI								
normal weight	-0.08	0.35	-0.01	0.97	-0.02	0.85	0.02	0.82
overweight	-0.13	0.11	0.04	0.64	0.05	0.55	0.19	0.16
obese	-0.03	0.85	0.26	0.05	0.24	0.08	0.08	0.36
Smoking								
no	-0.17	**0.027**	0.04	0.58	-0.0001	0.99	0.10	0.18
yes	0.05	0.56	0.08	0.30	0.11	0.15	0.07	0.47
Drinking								
no	-0.03	0.70	0.08	0.28	0.10	0.20	0.07	0.35
yes	-0.11	0.16	0.04	0.63	0.01	0.88	0.08	0.28
Diabetes								
no	-0.13	0.06	-0.02	0.83	-0.03	0.69	0.02	0.80
yes	-0.05	0.52	0.15	0.07	0.16	0.06	0.14	0.09
Hypertension								
no	0.01	0.89	0.04	0.63	0.05	0.48	0.03	0.70
yes	-0.21	**0.008**	0.09	0.28	0.06	0.47	0.12	0.14
Hyperlipidemia								
no	-0.06	0.33	0.02	0.77	0.02	0.69	0.05	0.44
yes	-0.21	0.13	0.29	**0.04**	0.26	0.07	0.23	0.10

Data were analyzed within subgroups categorized by age, sex, BMI, smoking status, alcohol consumption, diabetes mellitus, hypertension, and hyperdyslipidemia. Older adults were defined as those aged ≥ 60 years, while younger adults were those aged< 60 years; normal weight indicates 18.5 kg/m^2^≤ BMI< 24 kg/m^2^, overweight indicates 24 kg/m^2^ ≤ BMI< 28 kg/m^2^, and obesity indicates BMI ≥ 28 kg/m^2^. mtDNAcn, mitochondrial DNA copy number; BMI, body mass index. FT3/FT4, free triiodothyronine to free thyroxine ratio; TSHI, thyroid-stimulating hormone index; TT4RI, thyrotrophic thyroxine resistance index; TFQI, thyroid feedback quantile-based index. The bold values indicate p-values below 0.05, which are considered statistically significant.

### 3.3 Multiple linear regression analysis

To further explore the independent relationship between mtDNAcn and thyroid hormone sensitivity, a multiple linear regression analysis was conducted (**Table 3**). The FT3/FT4 ratio was significantly and negatively associated with mtDNAcn (β=-0.15, 95% CI-3.35 to -0.36, *P* =0.015). This association persisted even after adjusting for potential confounders, including age, sex, BMI, smoking and drinking use, and the presence of hypertension, diabetes, and hyperlipidemia.

**Table 3:** Relationships between thyroid parameters and mtDNAcn.

Variables	Model 1					Model 2				
	B	SE	β	t	*P*-value	B	SE	β	t	*P*-value
FT3	-0.098	0.06	-0.106	-1.629	0.104	-0.103	0.061	-0.11	-1.671	0.096
FT4	0.025	0.016	0.084	1.537	0.125	0.023	0.016	0.077	1.403	0.161
TSH	0.036	0.036	0.054	0.999	0.319	0.035	0.036	0.053	0.972	0.332
FT3/FT4	-1.860	0.754	-0.145	-2.465	**0.014**	-1.857	0.761	-0.145	-2.439	**0.015**
TSHI	0.106	0.064	0.088	1.642	0.101	0.100	0.064	0.084	1.554	0.121
TT4RI	0.003	0.002	0.073	1.352	0.177	0.003	0.002	0.071	1.313	0.190
TFQI	0.199	0.101	0.106	1.965	0.050	0.184	0.102	0.098	1.809	0.071

Data were analyzed using linear regression models. Model 1: adjusted for age, sex, and BMI; Model 2: adjusted for age, sex, BMI, drinking, smoking, diabetes, hypertension, and hyperlipidemia. Statistical significance was defined at *P*-value*<* 0.05. B, unstandardized beta coefficient; SE, standard error for unstandardized beta; β, standardized beta coefficient; t, t statistic; mtDNAcn, mitochondrial DNA copy number; FT3, free triiodothyronine; FT4, free thyroxine; TSH, thyroid-stimulating hormone; TSHI, thyroid-stimulating hormone index; TT4RI, thyrotrophic thyroxine resistance index; TFQI, thyroid feedback quantile-based index. The bold values indicate p-values below 0.05, which are considered statistically significant.

### 3.4 Subgroup-specific regression findings

**Figure 2:**
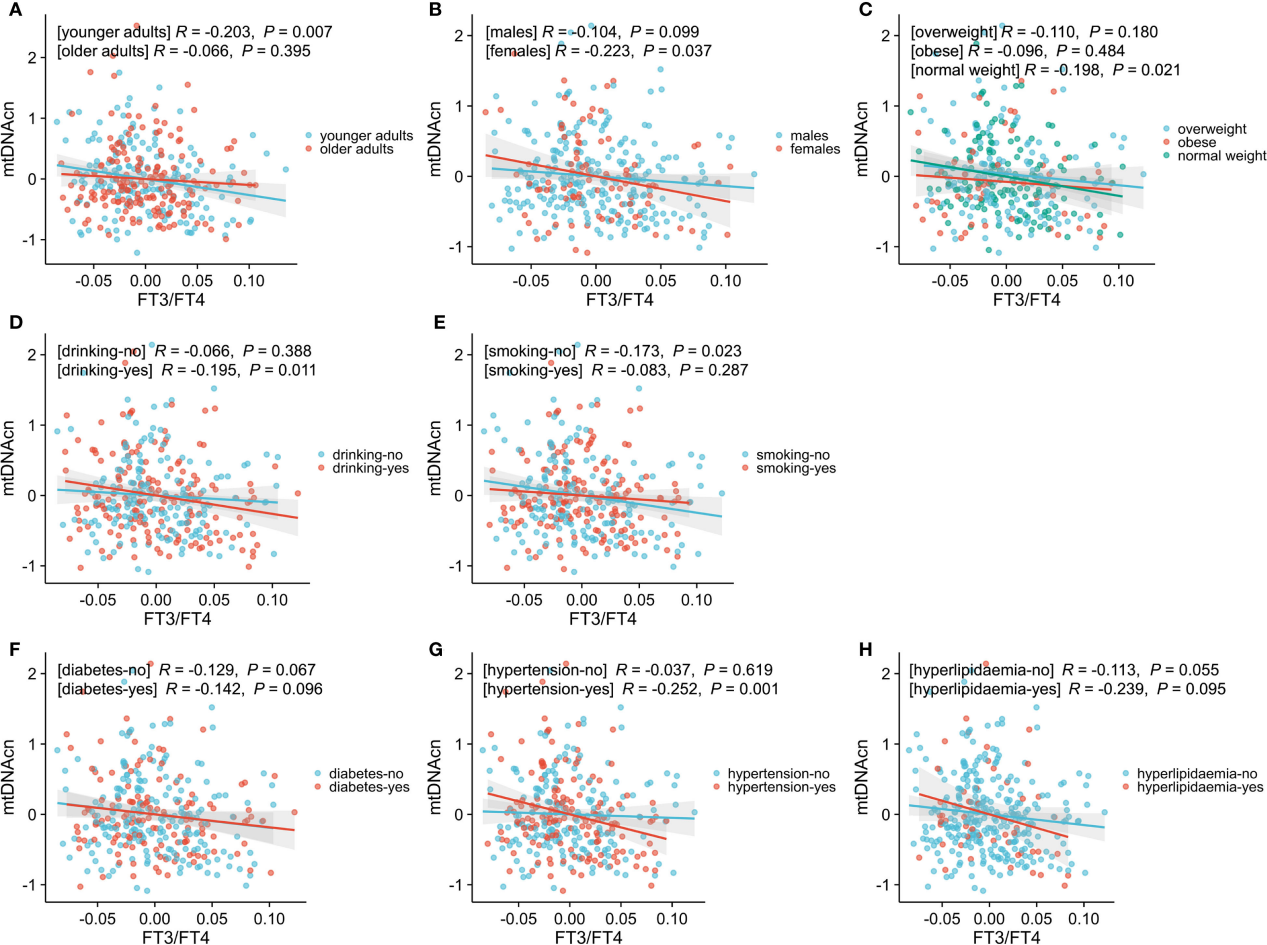
Associations between mtDNAcn and FT3/FT4, stratified by age **(A)**, sex **(B)**, BMI **(C)**, drinking history **(D)**, smoking history **(E)**, diabetes history **(F)**, hypertension history **(G)**, and hyperlipidemia **(H)**. Data were adjusted for these variables. Statistical significance was defined as a *P*-value< 0.05. Younger adults represent age < 60 years, while the older adults represent age ≥ 60 years; normal weight indicates 18.5 kg/m^2^≤ BMI< 24 kg/m^2^, overweight indicates 24 kg/m^2^ ≤ BMI< 28 kg/m^2^, and obesity indicates BMI ≥ 28 kg/m^2^. mtDNAcn, mitochondrial DNA copy number; FT3/FT4, free triiodothyronine to free thyroxine ratio; BMI, body mass index.

Partial regression plots were used to visualize adjusted associations between mtDNAcn and the FT3/FT4 ratio across various subgroups (**Figure 2**). The following significant negative associations were observed: 1) Age: the younger subgroup showed a significant inverse correlation, while no association was seen among older adults; 2) Sex: a significant inverse association was observed in females, but not in males; 3) BMI: the negative correlation was limited to participants with normal BMI; it was not present in overweight or obese individuals; 4) Alcohol Use: drinkers exhibited a significant inverse relationship, which was not observed in non-drinkers; 5) Smoking: the negative correlation was found in non-smokers but absent in smokers; 6) Hypertension: a significant inverse association was found in those with hypertension, but not in those without hypertension; 7) Diabetes and hyperlipidemia Subgroups: no significant correlations were observed in these subgroups.

## 4 Discussion

This study revealed a significant negative correlation between relative mtDNAcn and the FT3/FT4 ratio in a euthyroid population, even after adjusting for potential confounders. Specifically, this inverse relationship remained significant across subgroups, including younger adults, females, individuals with normal BMI, drinkers, non-smokers, and hypertensive patients. However, no significant correlation was found between mtDNAcn and central thyroid hormone sensitivity indices such as TSHI, TT4RI, or TFQI. These findings suggested that relative mtDNAcn levels were negatively associated with peripheral thyroid hormones sensitivity. Subgroup analyses further supported consistent correlations across different demographic and health statuses, offering new insights into the relationship between mtDNAcn and thyroid hormone sensitivity in euthyroid individuals.

The FT3/FT4 ratio is a well-established indicator of peripheral thyroid hormone sensitivity, reflecting the efficiency of peripheral tissues in converting T4 to T3 (1, 15). Reduced thyroid hormone activity, characterized by diminished tissue responsiveness, involves defects in both the transport of thyroid hormone across cell membranes and their subsequent metabolism and action (42). A lower FT3/FT4 ratio indicates reduced peripheral tissue sensitivity to thyroid hormones, which may lead to energy imbalance within cells. Additionally, thyroid hormones are critical regulators of mitochondrial function (43–45), with T3 playing a critical role in mitochondrial biogenesis. Studies have demonstrated that T3 can modulate mtDNA expression, either directly or indirectly through nuclear transcription factors like NRF1 and PCG1α (46). Both hyperthyroidism and hypothyroidism can affect mitochondrial activity, with hyperthyroidism generally associated with increased metabolic rates and enhanced mitochondrial energy expenditure (47, 48), while hypothyroidism leads to reduced metabolic rates and mitochondrial dysfunction (49, 50).

Precise quantification of mtDNAcn in cellular and tissue samples is essential for evaluating alterations in mitochondrial function and biogenesis. Quantitative methods for assessing mtDNAcn include Southern and Slot Blot, competitive PCR (cPCR), quantitative real-time PCR (qPCR), fluorescence *in situ* hybridization (FISH), and next-generation sequencing (NGS) technologies such as whole-genome sequencing (WGS) and whole-exome sequencing (WES) (21). Among these, Southern and Slot Blot and cPCR have been largely phased out. The qPCR is a technique that quantifies DNA amplification by monitoring fluorescence signals in real time, calculating mtDNAcn by comparing the amplification cycle thresholds (Ct values) values of mitochondrial genes to those of nuclear genes (51–53). FISH, though it enables direct visualization of mtDNA and provides valuable insights into its spatial organization and regulatory dynamics, is suitable for qualitative or semi-quantitative (e.g., nuclear dot counting) (54, 55). NGS methods estimate mtDNAcn through WGS by comparing the sequencing depth of mitochondrial and nuclear genomes, or through WES using “off-target” data (56–58). NGS, particularly WGS, offers significant advantages for large-scale studies, such as The Cancer Genome Atlas (TCGA), and holds potential for clinical standardization. Nevertheless, qPCR remains the most widely used technique for mtDNAcn quantification due to its simplicity, cost-effectiveness, and broad availability. Consequently, qPCR was utilized in this study for mtDNAcn assessment.

Our results suggested that decreased peripheral thyroid hormone sensitivity, as indicated by a lower FT3/FT4 ratio, was linked to higher mtDNAcn levels. This finding may reflect a negative feedback mechanism, where peripheral tissues reduce mitochondrial biosynthesis in response to excessive energy production. Previous studies have shown that thyroid hormones regulate mitochondrial biosynthesis and oxidative phosphorylation via nuclear receptors such as TRα and TRβ (39, 46). When thyroid hormones sensitivity diminishes, mitochondria may compensate by increasing mtDNAcn to sustain ATP production, thus promoting compensatory mitochondrial proliferation (36). Furthermore, reduced thyroid hormone sensitivity could lead to elevated oxidative stress, causing mtDNA damage. In response, cells may increase mtDNAcn to repair the damage and preserve mitochondrial function (59). In contrast to studies in hyperthyroid models that show increased mitochondrial biosynthesis through the activation of the PCG1α pathway, our findings suggested that different mechanisms regulate mitochondrial function in euthyroid individuals compared to pathological hyperthyroidism (59, 60).

Moreover, the negative correlation between the FT3/FT4 ratio and mtDNAcn was consistent across subgroups, including younger individuals, females, and those with normal BMI. This suggested that mitochondrial function in these groups was particularly sensitive to thyroid hormone regulation. In younger individuals, higher mitochondrial turnover rates may result in more pronounced regulation of peripheral hormone sensitivity (61). For females, estrogen might enhance mitochondrial biosynthesis in concert with thyroid hormones, strengthening the observed association (62, 63). Metabolically, individuals with normal weight typically show greater insulin sensitivity, and imbalances in the thyroid hormone-mitochondria axis may lead to more noticeable changes in mtDNAcn. In hypertensive individuals, endothelial mitochondrial dysfunction may further exacerbate this relationship (10, 64).

The clinical implications of our findings are noteworthy. The FT3/FT4 ratio, compared to FT3, FT4, or TSH, may serve as a more sensitive marker of thyroid hormone changes in euthyroid individuals. For example, previous research by wang et al. showed a positive association between FT3/FT4 ratio and grip strength, whereas TSH showed no such relationship (16). Similarly, elevated FT3/FT4 ratio had been observed in patients with non-alcoholic fatty liver disease, despite no changes in TSH levels (65). Moreover, the FT3/FT4 ratio had been linked to obesity, hypertriglyceridemia, and hypertension (3, 9, 66), suggesting its potential as an early indicator of metabolic disturbances in euthyroid individuals.

In contrast to the FT3/FT4 ratio, central thyroid hormone sensitivity indices (e.g., TT4RI, TSHI, TFQI) primarily reflect hypothalamic–pituitary–thyroid (HPT) axis function, and are involved in regulating metabolic and nervous system functions. Reduced central sensitivity to thyroid hormones had been associated with conditions such as obesity, diabetes, and metabolic syndrome (11, 67, 68). However, our findings showed no correlation between these indices and mtDNAcn, suggesting that central thyroid hormone sensitivity did not directly influence mtDNAcn in peripheral tissues. This indicated a tissue-specific regulation of thyroid hormone sensitivity that affects peripheral and central systems differently. Further research is needed to explore how thyroid hormone sensitivity may influence mitochondrial function.

A reduction in peripheral blood mtDNAcn had been linked to several diseases, such as metabolic syndrome, cardiovascular disease, type 2 diabetes, dementia, and future mortality risk (27, 29–32). Studies had also shown that mtDNAcn in thyroid patients was lower than in healthy individuals. Notably, patients with malignant thyroid nodules had higher mtDNAcn levels compared to those with benign nodules. Furthermore, increased mtDNAcn had been suggested as an independent risk factor for thyroid cancer (36, 64). These findings suggested that elevated mtDNAcn might be an adaptive response to mitochondrial stress (69), as mitochondria increase mtDNAcn to meet increased energy demands. In this context, the negative relationship between peripheral thyroid hormone sensitivity and mtDNAcn could reflect a compensatory mechanism, particularly in groups with heightened metabolic demand or hormonal sensitivity. This compensation could involve the upregulation of TFAM and PGC-1α signaling pathways to enhance mitochondrial biosynthesis, as part of the cellular adaptation to altered thyroid hormone sensitivity.

Importantly, this compensatory response may be driven by the established role of thyroid hormones in regulating mitochondrial biogenesis through the PGC-1α/TFAM pathway, where TFAM stabilizes mtDNA and promotes replication (39). In individuals with impaired thyroid hormone sensitivity, peripheral tissues may upregulate mitochondrial biogenesis as a compensatory mechanism to counteract reduced mitochondrial function, leading to increased mtDNAcn. This adaptive response, involving elevated TFAM expression, may serve to preserve cellular energy production despite suboptimal thyroid hormonal signaling, highlighting the significance of mitochondrial compensation in euthyroid individuals.

Our study has some limitations. As a cross-sectional study, we cannot draw conclusions about causality between thyroid hormone sensitivity and mtDNAcn. Longitudinal studies are needed to assess this relationship over time. Additionally, while our study provides insights into the link between mtDNAcn and thyroid hormone sensitivity, the underlying molecular mechanisms remain unclear. Future research should explore these mechanisms through animal and cellular models. Lastly, whether these findings can be translated into diagnostic tools or clinical interventions requires further validation.

## 5 Conclusion

In conclusion, our study suggested that decreased mtDNAcn was associated with impaired peripheral sensitivity to thyroid hormones in euthyroid individuals, with variations across age, sex, BMI, smoking status, and hypertension. These findings highlight the complex interconnection between thyroid hormone sensitivity and mitochondrial function, warranting further investigation to elucidate the underlying mechanisms.

## Data Availability

The raw data supporting the conclusions of this article will be made available by the authors, without undue reservation.
